# Development of an inventory to assess perceived barriers related to PKU treatment

**DOI:** 10.1186/s41687-020-00194-w

**Published:** 2020-05-01

**Authors:** Katia Irie Teruya, Eduardo Remor, Ida Vanessa Doederlein Schwartz

**Affiliations:** 1 Institute of Psychology, Universidade Federal do Rio Grande do Sul. Rua Ramiro Barcelos, 2600 (sala 219), Porto Alegre, Rio Grande do Sul 90035003 Brazil; 2 Department of Genetics, Universidade Federal do Rio Grande do Sul, Porto Alegre, Rio Grande do Sul Brazil

**Keywords:** Phenylketonuria, Phenylalanine, Perceived barriers, Treatment, Measurement

## Abstract

**Background:**

According to studies of phenylketonuria (PKU), the Brazilian population’s metabolic control shows unsatisfactory indexes from childhood. Research on patients’ perceived difficulties or barriers to adherence to treatment can help us to comprehend how these outcomes are associated. The present study aimed to: (1) describe the development of an inventory for identifying the most frequent and relevant perceived barriers to PKU treatment from the perspective of patients, caregivers, and healthcare professionals; (2) evaluate certain psychometric characteristics of the new measure; and, (3) explore potential predictors (sociodemographic and medical characteristics) that may contribute to increasing the number of perceived barriers and examine whether the number of barriers is associated with the degree of adherence shown by the patient.

**Results:**

Participants in the study were 23 patients with PKU (*M* age = 18.0 years; *SD* = 7.3; range 6 to 34 years; 69% early-treated) in classical (*n* = 11) and mild (*n* = 12) form, and 11 caregivers. The inventory, developed to ascertain perceived barriers to treatment, was completed by patients (≥ 13 years) and caregivers of patients aged 6 to 17 years. Analyses were conducted to investigate whether barrier inventory scores were associated with adherence to treatment as measured by phenylalanine levels in patients’ medical records. Scores on the inventory differed across the patient age groups: adolescents had lower scores (i.e. reported fewer barriers) compared with those of adults (*U* = 8.000, *p* = 0.008); patients with better recent metabolic control also reported fewer perceived barriers than did patients with poor adherence (*U* = 20.000, *p* = 0.009); and the number of perceived barriers was positively associated with recent blood phenylalanine concentration (Kendall’s tau_b_ = 0.41; *p* = 0.001).

**Conclusions:**

These results suggest that the inventory has merit in assessing perceived barriers and support the need for further research on barriers perceived by PKU patients.

## Introduction

Phenylketonuria (PKU; OMIM 261600) is an inherited, autosomal recessive disorder caused by a deficiency in the enzyme phenylalanine hydroxylase (PAH) [1]. Phenylalanine hydroxylase converts phenylalanine (Phe) into tyrosine and requires the cofactor tetrahydrobiopterin (BH4), molecular oxygen, and iron to do so [2]. Deficiency in phenylalanine hydroxylase activity results in higher concentrations of phenylalanine in the blood and may lead to a range of symptoms, from eczematous rash to motor deficits and seizure episodes [1, 3].

Phenylalanine is a fairly common amino acid present in foods such as meat, fish, milk, cheese, eggs, nuts, seeds, products with flour, and soy [4]. Thus, available treatments, which aim to avoid the deleterious effects of excess phenylalanine in the blood, consist of two dietary recommendations: patients are advised not to ingest any food containing phenylalanine in its composition, and to consume protein substitutes, called medical food, at least three times per day [4]. Medical foods are available in the form of phenylalanine-free or phenylalanine-reduced nutritionally complete drink mix powders that contain energy, amino acids, and vitamins [5].

Examining adherence rates to PKU treatment reveals a recurring finding in studies conducted in different years and countries: an increase in non-adherence as patients become older [6–11]. For example, a UK study from 2004 showed that on average 17% of children had above the recommended range of plasma Phe, increasing to 75% when the patient was 20 years old [11]. In 2017, a study conducted in the US showed a similar relationship between rates of non-adherence among children (12% in patients of 0–4 years of age) and adult patients (67% showed Phe levels higher than the target values) [8].

Unlike many countries, non-adherence among Brazilian phenylketonuria patients is a phenomenon that is not restricted to a specific age group, nor is it related to the transition from childhood to adulthood. According to the few studies carried out in Brazil, adherence rates already present unsatisfactory indexes during patients’ childhood. One study found that 77.4% of patients under 13 years of age had blood phenylalanine levels above treatment goals [12]. In another survey of patients aged six to 18 years, the non-adherence rate was 68.6% [13].

Several aspects have been mentioned as potential contributors to these rates of adherence: social pressures that hinder the integration of the individual with PKU into society, a lack of time to adjust to dietary requirements, financial burden as a result of the high cost of special foods, unfamiliarity with phenylalanine levels in foods, poor adherence to protein substitutes, conflicting ideologies about illness and treatment, poor healthcare professional/career/patient relationships, family culture, poor social and/or family support, lack of symptoms, and negative attitudes toward the condition or dietary treatment, among others [14–16].

Studies of decision-making processes concerning health behavior have mentioned the influence of an individual’s perception of barriers to achieving a goal as being relevant [17]. Under conditions as diverse as pain management [18, 19], physical mobility [20], breast self-examination [21], diabetes [22], cystic fibrosis [23], human papillomavirus vaccination [17], hypertension [24], mammography screening [25], HIV infection [26], and hemophilia [27], it has been observed that individual experiences and decisions are influenced by perceived barriers, affecting their adherence to treatment in the case of chronic diseases.

In the 1970s, perceived barriers were already being pointed out as an important factor that could influence health outcomes, as suggested by the Health Belief Model (HBM) [28]. Financial burden, inconvenience, pain, and feelings of embarrassment could all be considered costs, and all costs perceived by an individual can be seen as barriers if they reduce the likelihood of that individual adopting a habit. Moreover, individual factors—such as demographic (e.g. age, sex, ethnicity), psychosocial (e.g. personality, peer pressure), and structural (e.g. knowledge about the disease, time of living with diagnosis) variables [29]—can turn an event into a barrier to healthy behavior. Becker et al. [20] have also mentioned the relevance of a comprehensive approach when studying such difficulties in a given population.

Assessment tools for the perceived barriers construct have already been proposed for health conditions such as melanoma [30], heart failure [31], diabetes [32], and HIV infection [33]. However, to the best of our knowledge there is no such measure for phenylketonuria. For this reason, based on evidence about the influence of perceived barriers on health decisions in other contexts, we believe that the development of structured forms of evaluation for this construct can help to advance knowledge in the field of adherence to phenylketonuria treatment too. Furthermore, such a tool could be used to assess whether perceived barriers vary according to certain individual characteristics, such as age, sex, parental education, and/or others and, therefore, whether certain groups are more susceptible to experiencing more barriers to treatment.

The present study aimed to: (1) describe the development of an inventory for identifying the most frequent and relevant perceived barriers to PKU treatment from the perspective of patients, caregivers, and healthcare professionals; (2) evaluate certain psychometric characteristics of the new measure; and, (3) explore potential predictors (sociodemographic and medical characteristics) that may contribute to increasing the number of perceived barriers and examine whether the number of barriers is associated with the degree of adherence shown by the patient.

## Methods

### Initial development of the phenylketonuria perceived barriers to treatment inventory

#### Step 1. Item construction and development

- Literature review: In order to identify experiences relating to phenylketonuria treatment, a literature review was performed by searching the electronic databases PsycInfo, Medline, Scielo, and IndexPsi. A time period was not specified because of the rare incidence of the disease in the population [3]. Key words “phenylketonuria” and “adherence” were used as a first step in the search, but articles were chosen only if they contained content about the experiences of patients and their families in relation to the condition and its treatment (see references reviewed at Table 1). An iterative approach was used, and when new references failed to provide further relevant information the review was concluded.

(b). Qualitatively, the articles’ contents were collected, classified, rephrased, and labeled into topics according to five interacting dimensions that according to World Health Organization can affect adherence behavior [43]. Each of the five dimensions—Social/economic factors, Healthcare team and system-related factors, Condition-related factors, Therapy-related factors, and Patient-related factors—was represented by one or more topics (see Table 2).

(c). Lastly, each topic was translated into a sentence reflecting the patient’s or caregiver’s point of view (e.g. “Difficulties about what to order from the menu when eating out” was represented by the item: “I get a little confused when I go to a restaurant. I do not know what I can order.”). A topic could be represented by one or more items, with the result that 27 items were developed for inclusion in the initial version of the inventory.

**Table 1:** Articles chosen from the literature to guide the questionnaire item construction

Authors (year of publication)	Article’s title	Journal, vol., pages
Bik-Multanowski et al. (2008) [34]	Quality of life in noncompliant adults with phenylketonuria after resumption of the diet.	Journal of Inherited Metabolic Disease, 31, 415–418.
Bilginsoy et al. (2005) [35]	Living with phenylketonuria: Perspectives of patients and their families.	Journal of Inherited Metabolic Disease, 28, 639–649.
Bosch et al. (2015) [36]	Assessment of the impact of phenylketonuria and its treatment on quality of life of patients and parents from seven European countries.	Orphanet Journal of Rare Diseases, 10, 80–94
Di Ciommo et al. (2012) [37]	Living with Phenylketonuria from the point of view of children, adolescents, and young adults: a qualitative study*.*	Journal of Developmental and Behavioral Pediatrics, 33, 229–235.
Diesen et al. (2015) [38]	Betwixt and between being healthy and ill: the stigma experienced by young adults with phenylketonuria.	Scandinavian Journal of Disability Research, 17, 321–334.
Ievers-Landis et al. (2005) [39]	Situational analysis of dietary challenges of the treatment regimen for children and adolescents with phenylketonuria and their primary caregivers.	Developmental and Behavioral Pediatrics, 26, 186–193.
Kemper et al. (2010) [40]	Perspectives on Dietary Adherence among Women with Inborn Errors of Metabolism.	Journal of American Dietetic Association, 110, 247–252
MacDonald et al. (2010) [15]	The reality of dietary compliance in the management of phenylketonuria.	Journal of Inherited Metabolic Disease, 33, 665–670.
MacDonald et al. (2012) [14]	Adherence Issues in Inherited Metabolic Disorders Treated by Low Natural Protein Diets.	Annals of Nutrition and Metabolism, 61, 289–295.
Sharman et al. (2013) [41]	Qualitative Analysis of Factors Affecting Adherence to the Phenylketonuria Diet in Adolescents.	Clinical Nurse Specialist, 27, 205–210.
Vegni et al. (2009) [42]	How individuals with phenylketonuria experience their illness: an age-related qualitative study.	Child: care, health and development, 36, 539–548.
Vieira et al. (2015) [12]	Adherence to Treatment of Phenylketonuria: A Study in Southern Brazilian Patients.	Journal of Inborn Errors of Metabolism & Screening, 3, 1–7.

**Table 2:** The five dimensions of adherence and topics related to PKU treatment according to literature

WHO Adherence Dimensions	Topics (reference)
Social/Economic-related factors	Poor social or family support; family skills and dynamics [15, 40, 44]
Health care team and system-related factors	Poor access to the health system [15]
	Failure in receiving medication from public sources [12]
	Feeling of detachment in relation to the medical staff [42]
Condition-related factors	Lack of perceived disease symptoms [15, 40]
	Dealing with diagnosis as a potential risk and not as an established disease [37, 38]
Therapy-related factors	Time constraints to prepare meals [15, 35, 41]
	Difficult in preparing adequate, varied and nutritious meals [15, 34]
	Difficulties about what to order from the menu when eating out [15, 39]
	Low palatability of medical food [14, 36, 39, 40]
	High level of dietary restrictions [12]
Patient-related factors	Lack of ability to cope with food temptation [36, 40, 41]
	Fear of being treated differently by others, fear of stigma [15, 36, 38, 40]
	Feeling ashamed to talk about diagnosis with others [41]
	Restriction on social life [35, 36]
	Poor knowledge about the disease [15, 39]
	Poor knowledge about the treatment [15]
	Lack of motivation to follow the treatment [15]
	Denial of condition [15]
	Lack of conviction that poor compliance will have adverse effects [15]

#### Step 2. Content and face validity

The second step involved the assessment of items by experts. Three phenylketonuria specialists (i.e. a physician, a nurse, and a nutritionist) were invited to help with the inventory’s construction, providing suggestions on items’ relevance and clarity. Fifteen of the 27 items achieved 100% agreement among these experts in terms of content relevance, nine items were viewed as relevant by two of the specialists, and three items were considered relevant by one. No item was considered irrelevant by all three experts, so no item was removed from the initial version of the inventory. Seven items achieved 100% agreement among the experts in terms of clarity and therefore remained unchanged. Fifteen items that did not achieve 100% agreement among the experts were modified. Modifications were made based on the contributions of the experts and researchers. Five items remained in their original form because the researchers were unable to come up with satisfactory rewording and because of the exploratory nature of the study it was decided to retain them. Although, no patients were consulted during this phase because of the limited number of participants available, during data collection phase the inventory included an open ended question allowing the participant to add a particular perceived barrier to treatment that was not listed in the inventory (see supplementary material). In addition, researcher interviewing patients was trained to be very sensitive and open to get feedback from patients regarding the assessment protocol, this posture had brought valuable information regarding the barriers assessment inventory. At the conclusion of this step, the draft version of the inventory was complete and ready for to be used for data collection.

### Study setting and design

After developing the pool of items to be included in the inventory, the present study assessed phenylketonuria patients followed up through a Medical Genetics Service at a University Hospital between May 2018 and January 2019. This was a cross-sectional study involving the collection of retrospective clinical information and the use of prospective patient-reported outcomes. Since almost all the patients lived in another city, far from the hospital, the study protocol was conducted on the same day on which the patients’ consultation with the health team took place. Because phenylketonuria is a rare disease [45], a sample calculation was not performed. Instead, we tried to assess as many patients as possible. Hence, we carried out non-probabilistic, consecutive sampling, based on patients’ availability and desire to take part in the survey [46].

### Participants

Patients aged six years or older and caregivers of patients aged between 6 and 17 years were invited to participate in the study. All participating patients were required to have a diagnosis of either classical, mild or undefined type phenylketonuria and to be on a protein-restricted diet supplemented by Phe-free amino acid formula (medical food). Classical phenylketonuria was defined as having pre-treatment blood Phe levels repeatedly of > 1200 μmol/l (> 20 mg/dL); mild phenylketonuria was characterized by pre-treatment blood Phe levels in the range of 600–1200 μmol/l (10–20 mg/dL); and undefined type phenylketonuria reflected unavailable data on Phe levels at diagnosis [1].

In order to classify patients according to early- or late-treatment onset, information on the date of initiation of treatment was collected. A diagnosis is considered to be late if a child is diagnosed after the age of 3 months [3], so we used the same parameter to classify patients according to the beginning of their treatment. Exclusion criteria for the current study were as follows: being younger than 6 years old, the presence of severe chronic or disabling diseases unrelated to PKU, the inability (of either patients or caregivers) to understand the study questionnaires, being allowed to follow an unrestricted diet, and having an irregular follow-up during the study period (i.e., miss appointments). During the study period, the hospital had 88 attending patients. However, 13 of them were younger than 6 years of age, three had another PKU-associated disease (one with bipolar disorder, one with Down syndrome, and one with epileptic encephalopathy), 19 had a high level of impaired general development meaning that they were unable to understand the questionnaires, eight were considering to have an irregular follow up (they failed to show up at the hospital during the study period), and one was on an unrestricted diet. Therefore, of the 88 patients who were followed up, 44 patients met the inclusion criteria and did not fall foul of the exclusion criteria. Nevertheless, once the data collection began eight no longer agreed to participate in the study, most of them citing a lack of time; one could not participate because she had not been able to sleep the night before; and 12 could not be contacted during the research period because of scheduling issues.

### Variables and measures

#### Sociodemographic characteristics

Data on sociodemographic characteristics (e.g. age, sex, caregivers’ education level, and socioeconomic status) were provided by the patient or family member and recorded on a sheet.

#### Medical data and phenylketonuria clinical variables

Retrospective and prospective information was collected from patients’ medical charts. The median number of examinations performed in the last 12 months and the most recent phenylalanine level prior to the study were used to measure patient compliance.

#### Self-rated level of knowledge about the disease and dietary treatment, and perceived adherence to medical treatment^1^

Were collected by means of three, single item, visual analogue scales (VAS)—i.e. *Disease knowledge* – VAS (Patients: When you compare yourself to other phenylketonuria patients, how much would you say you know about the disease?; Proxy: When you compare yourself to others parents of patients with PKU, how much would you say you know about the disease? Non-knowledge to Excellent knowledge); *Dietary knowledge* – VAS (Patient: When you compare yourself to other phenylketonuria patients, how much would you say is your knowledge about the diet?; Proxy: When you compare yourself to the other parents of patients with PKU, how much would you say is your knowledge about the diet? Non-knowledge to Excellent knowledge); and *Perceived adherence* – VAS (Patient: When you compare yourself to other phenylketonuria patients, how much would you say your behaviors are in agreement with the medical treatment prescribed?; Proxy: When you compare yourself to other parents of patients with PKU, how much would you say your behaviors (your child’s behaviors) are in agreement with the medical treatment prescribed? Disagreement to Agreement) — reproduced graphically by a 100 mm line and printed on a sheet of paper.

#### Information concerning perceived barriers to adherence to phenylketonuria treatment

This was collected using the Perceived Barriers to Phenylketonuria Treatment Inventory developed for this study. This self-report tool comes in two different versions: one for patients aged 13 years and above (Perceived Barriers to Phenylketonuria Treatment Inventory - Patient version), the other for caregivers of patients aged six to 17 years (Perceived Barriers to Phenylketonuria Treatment Inventory - Caregiver version). Includes 27 statements related to barriers to treatment, were the participant (patient or caregiver) can select the statement that best represents their experience related to treatment. The inventory includes, at end of the instrument, an open ended question allowing the participant to describe a particular perceived barrier to treatment that was not listed in the inventory.

#### Intellectual and cognitive ability (IQ)

This was assessed using the Wechsler Abbreviated Scale of Intelligence (WASI [47];). The WASI is the most widely used IQ test for both adults and older adolescents in the world. It includes four subtests: Vocabulary, Block Design, Similarities, and Matrix Reasoning and allows Full-Scale IQ, Verbal IQ, and Performance IQ to be measured.

### Study protocol and procedures

The study protocol began with the invitation to take part in the study, providing information about the study and requesting the patient’s written consent. For pediatric patients, parents’ written consent and the assent of the children were requested. After that, data on sociodemographic characteristics were provided by the patient or family member. Next, self-rated level of knowledge about the disease (Disease knowledge -VAS) and its dietary treatment (Dietary knowledge - VAS), and perceived adherence to medical treatment (Perceived adherence -VAS) were collected by means of three, single visual analogue scales. The scales were completed by the person in charge of managing the patient’s treatment—either the patient or the caregiver—according to the agreement between them. Information concerning perceived barriers to adherence to phenylketonuria treatment was also collected from either the patients aged 13 years and older (PKU Perceived Barriers to Treatment Inventory - Patient version) or the caregivers of patients aged six to 17 years (Phenylketonuria Perceived Barriers to Treatment Inventory - Caregiver version).

Lastly, in order to estimate phenylketonuria patients’ intellectual and cognitive ability (IQ), the Wechsler Abbreviated Intelligence Scale (WASI) test was used. A trained psychologist administered the tests in accordance with the instructions in its manual, and age-referenced normative data from the manual validated for the Brazilian population were used to generate an estimated IQ.

Participants ideally completed the protocol in a single session of approximately 50 min. However, in some cases it took two sessions: one for the patient-reported outcome measures and another for the WASI. Once the study protocol had been completed, medical data and information were taken from the patients’ medical records. In accordance with van Wegberg et al.’s [3] directions, adherence was classified as good when the Phe median values of a patient were between 2 and 6 mg/dL (120–360 μmol/L) in patients up to 12 years of age, and between 2 and 10 mg/dL (120–600 μmol/L) in patient aged 12 years or older.

### Ethics approval and consent to participate

All aspects of the project and study were conducted in compliance with the Code of Ethics of the World Medical Association (Declaration of Helsinki). The study was approved by the Research Ethics Review Committee of the University Hospital (CAAE: 88184518.6.0000.5327). Adult patients, patients aged ≥ 6 years of age and parents or legal guardians of patients aged between six and 17 years were entered into the study only following their agreement and signing of their informed consent after they had been given information on the goals of the study and other relevant information. The assent of the children themselves was also requested for all pediatric patients.

### Data processing and statistical analysis

The data were examined for missing information, outliers, and normality. Because some of the variables did not follow a normal distribution, they were expressed in terms of medians and interquartile range. The following measures were considered continuous variables: age, age of diagnosis, intelligence measurements (Full-Scale IQ, Verbal IQ, and Performance IQ), phenylalanine measures in mg/dL, VAS scores (Disease knowledge-VAS, Dietary knowledge -VAS, Perceived adherence-VAS), and Perceived Barriers to Phenylketonuria Treatment Inventory scores. Due to both the non-normality of some variables and the sample size, non-parametric tests were used. The Mann-Whitney test was used to compare the two groups in relation to each variable. Kendall’s Tau-b correlation coefficient was used to analyze the association between continuous variables. Fisher’s Exact test was used to compare the groups in terms of the non-continuous variables, such as sex, current age (< 18 years of age and ≥ 18 years of age) classifications of PKU (classical or mild), caregivers’ education (≤ 4 years of education, ≥ 5 years of education), onset of treatment (≤ 3 months of age, > 3 months of age), and adherence status (good, poor). Statistical significance was accepted at *p* < 0.05, and statistical analyses were performed using SPSS 18 version software (SPSS, Inc., Chicago, IL, USA).

## Results

### Characteristics of study participants

Twenty-three patients took part in the study. The mean age was 18.1 years (*SD* = 7.4; age range = 6 to 34 years). The mean age of patients under 18 years of age was 12.6 years (*SD* = 4.8) and among the adult patients 23.1 years (*SD* = 5.6). Approximately 26% of patients had no siblings, 52% had one or two, and 22% had three or more. One patient had one sibling with phenylketonuria and one had two siblings with the same diagnosis. The median income reported was 1.5 times the minimum wage for both mothers and fathers. See details in Table 3.

**Table 3:** Demographics characteristics of patients with Phenylketonuria in the study, according to age group

Variable	Total sample *n* (%)	< 18 years of age (*n* = 11) *n* (%)	≥ 18 years of age (*n* = 12) *n* (%)	*p*
Sex				1.000^a^
Woman	9 (39.1)	4 (44.4)	5 (55.6)	
Man	14 (60.9)	7 (50.0)	7 (50.0)	
Mothers’ level of education				0.193 ^a^
4 years or less	8 (34.8)	2 (25.0)	6 (75.0)	
5 years or more	15 (65.2)	9 (60.0)	6 (40.0)	
Fathers’ level of education (*n* = 19)^§^				0.319^a^
4 years or less	6 (31.6)	2 (33.3)	4 (66.7)	
5 years or more	13 (68.4)	9 (69.2)	4 (30.8)	
PKU Classification^¥^				0.414^a^
Classical	11 (47.8)	4 (36.4)	7 (63.6)	
Mild	12 (52.2)	7 (58.3)	5 (41.7)	
Beginning of treatment				0.667^a^
≤ 3 months of age	15 (65.2)	8 (53.3)	7 (46.7)	
> 3 months of age	8 (34.8)	3 (37.5)	5 (62.5)	
Current Phe Adherence(*n* = 22)^£^				0.198^a^
Good	12 (54.5)	8 (66.7)	4 (33.3)	
Poor	10 (45.5)	3 (30.0)	7 (70.0)	
Current Median Phe Adherence (*n* = 22)^£^				0.670^a^
Good	10 (45.5)	6 (60.0)	4 (40.0)	
Poor	12 (54.5)	5 (41.7)	7 (58.3)	
Current Phe level (n = 22)^£^ mg/dL *Md* [*IQR*]	8.8 [6–14.3]	7.3 [5.9–8.9]	13.2 [7.4–17.3]	0.017^b^
Current Median Phe level (n = 22)^£^ mg/dL *Md* [*IQR*]	8.3 [6.9–14.4]	7.8 [5.6–8.3]	13.2 [7.2–18.7]	0.039^b^

*Notes: Phe* Phenylalanine; Level of education = years of schooling. Current Phe level = Phe level collected prior to the study. Current Median Phe level = median of Phe level collected in the last 12 months prior to the study. § Four patients did not answer the question on the paternal educational achievement. ¥ One patient with Undefined PKU type was classified as Classical PKU. £ One patient had no information regarding recent adherence

^a^Fisher’s Exact test

^b^Mann-Whitney test

### Perceived barriers to phenylketonuria treatment

During completion of the Phenylketonuria Perceived Barrier to Treatment Inventory, we observed that one sentence was being understood in two different ways by respondents. When questioned about what they had understood by the content of item 9 (i.e. “Having the disease does not change anything in my life”), some respondents related it to adaptive coping; that is, they did not associate the item with a sense of denial of the diagnosis as was the item’s intended meaning. On the other hand, some respondents did interpret it as per its intended meaning. Because of this ambiguity, item 9 was excluded from the analysis. Item 11 (i.e. “I believe that following or not following the diet makes no difference to my health”) was also excluded, because it was not mentioned by any participant. Given its content, it may be that the social desirability factor was responsible for its zero score.

Scores on the Phenylketonuria Perceived Barrier to Treatment Inventory differed across patient age groups: adolescents (13–17 years of age) had a lower score (*M* = 4.0; *SD* = 2.5, *Mdn* = 4, *IQR* = 1.8–5.8) compared with that of adults (18 years of age or older) (*M* = 9.7; *SD* = 4.2; *Mdn* = 9, *IQR* = 7.3–11.8) (*U* = 8.000, *p* = 0.008). The mean PKU Perceived Barriers to Treatment Inventory score for caregivers (parents of patients aged 6 to 17 years) was similar to that of the adolescent group (*M* = 5.0; *SD* = 2.5; *Mdn* = 4.0, *IQR* = 4.0–6.0). Eleven items in the inventory (44%) were not endorsed by any of the adolescents in the study, while those in the caregiver’s group did not endorse four (16%) of the 25 items. Among the adult patients, all items were endorsed by at least some of the respondents. Frequencies for the 25 items by group are presented in detail in Table 4.

**Table 4:** Frequency of responses to perceived barrier to Phenylketonuria treatment by groups of adolescents, adult patients and caregivers of patients (6 to 17 y.o)

Phenylketonuria Perceived Barrier to Treatment Inventory Patient version	Phenylketonuria Perceived Barrier to Treatment Inventory Caregiver version	(f) % agreement to item. Patients 13 to 17 years old (*n* = 6)	(f) % agreement to item. Patients 18 years of age or older (*n* = 12)	(f) % agreement to item. Caregivers of patients 6 to 17 y.o. (*n* = 11)
1. I am afraid that people will treat me differently or reject me if they know that I have the disease.	1. I am afraid that people will treat my child differently or reject him/her if they know that he/she has the disease.	(0) 0	(6) 50	(2) 18.2
2. Sometimes I hide from people that I have the disease.	2. Sometimes I hide from people that my child has PKU.	(0) 0	(5) 41.7	(1) 9.1
3. Sometimes I feel ashamed to tell people I have the disease.	3. Sometimes I feel ashamed to tell people my kid has PKU.	(0) 0	(2) 16.7	(0) 0
4. Although I feel like going, I don’t go to some parties or family events because I know that there will be food that I cannot eat.	4. Although we want to go to a party or family event, we don’t go because we know that there will be food that he/she cannot eat.	(0) 0	(5) 41.7	(3) 27.3
5. I have questions about what the disease is, what causes it, how it can harm me.	5. I have questions about what the disease is, what causes it, how it can harm him/her.	(0) 0	(5) 41.7	(1) 9.1
6. I do not understand what can happen if I do not get the treatment.	6. I do not understand what can happen if my child kid doesn’t follow the treatment.	(0) 0	(3) 25	(2) 18.2
7. I have questions about what I can eat, which food is forbidden, which one is allowed and how to control the diet.	7. I have questions about what my child can eat, which food is forbidden, which one is allowed and how to control the diet.	(0) 0	(3) 25	(2) 18.2
8. I often have no desire to follow the diet.	8. I frequently realize that he/she doesn’t want to follow the diet.	**(3) 50**	**(7) 58.3**	(3) 27.3
10. I believe that the disease can not harm my health.	10. I believe that the disease can not harm his/her health.	(1) 16.7	(2) 16.7	(1) 9.1
12. I feel that I do not have people to count on to help me follow the treatment.	12. I feel that I don’t have people to count on to help me with my child’s treatment.	(0) 0	(2) 16.7	(1) 9.1
13. People in my family say different things about the treatment and I do not know what to do.	13. Sometimes I realize we say different things about the treatment to him/her and we don’t know what to do.	(0) 0	(3) 25	(1) 9.1
14. I frequently have no time to prepare my meals.	14. I frequently have no time to prepare his/her meals.	(1) 16.7	(3) 25	(1) 9.1
15. Planning and preparing meals take a lot of my time on a daily basis and so I cannot fully follow the diet.	15. Planning and preparing meals takes a lot of my time on a daily basis and so I cannot make my child fully follow the diet.	(1) 16.7	(5) 41.7	(1) 9.1
16. I get a bit confused when it comes to deciding what to cook, what ingredients I can use.	16. I get a little bit confused when it comes to deciding what to cook, what ingredients I can use.	(1) 16.7	(4) 33.3	(2) 18.2
17. I get a little confused when I go to a restaurant. I do not know what I can order.	17. I get a little confused when we go to a restaurant. I do not know what we can order.	(1) 16.7	(4) 33.3	(1) 9.1
18. Sometimes I cannot resist and I eat foods I know are forbidden.	18. Sometimes I cannot resist and allow him/her to eat foods I know are not allowed.	(2) 33.3	**(8) 66.7**	(3) 27.3
19. It would be easier to take the formula if it had a better taste.	19. It would be easier to make him/her ingest the formula if it had a better taste.	**(3) 50.0**	**(11) 91.7**	**(8) 72.7**
20. I think the diet is very strict. If I could eat a broader range of food, it would be easier.	20. I think the diet is very strict. If he/she could eat a broader range of food, it would be easier.	(0) 0	**(11) 91.7**	**(7) 63.6**
21. I do not think I have a disease. I just need to take care of my meals.	21. I do not think my kid has a disease. He/she just needs to take care of what he/she eats.	**(3) 50.0**	**(7) 58.3**	(4) 36.4
22. I do not feel anything when I don’t follow the diet and that makes me not worry about the disease.	22. I can’t see any difference in my child when he/she doesn’t follow the diet and that makes me not to worry about the disease.	(1) 16.7	(5) 41.7	(2) 18.2
23. I notice that the medical staff treats me differently, as if I might not be able to follow the treatment.	23. I notice that the medical staff treats us differently, as if my child and I might not be able to follow the treatment.	(0) 0	(2) 16.7	(0) 0
24. I struggle to follow the treatment, but I feel that the medical team does not believe it.	24. We struggle to follow the treatment, but I feel that the medical team does not believe us.	(1) 16.7	(2) 16.7	(0) 0
25. I feel physicians do not believe what I say and think I do not follow what they instruct me to do.	25. I feel physicians do not believe what we say and they think we do not follow what they instruct us to do.	(1) 16.7	(2) 16.7	(0) 0
26. The distance from my house to the hospital makes it more difficult to attend the medical appointments.	26. The distance from our house to the hospital makes it more difficult to attend the medical appointments.	**(4) 66.7**	(2) 16.7	(4) 36.4
27. There are months when the formula is not delivered.	27. There are months when the formula is not delivered.	(1) 16.7	(5) 41.7	(5) 45.5

*Notes.* The items were translated from the Brazilian Portuguese to English to the present paper. Following the guidelines from the International Test Commission, researchers interested in using the inventory should contact the authors to obtain the complete version of the instrument, scoring instructions and proper authorization for use

Items mentioned by ≥50% of participants are printed in bold font

**Fig. 1:**
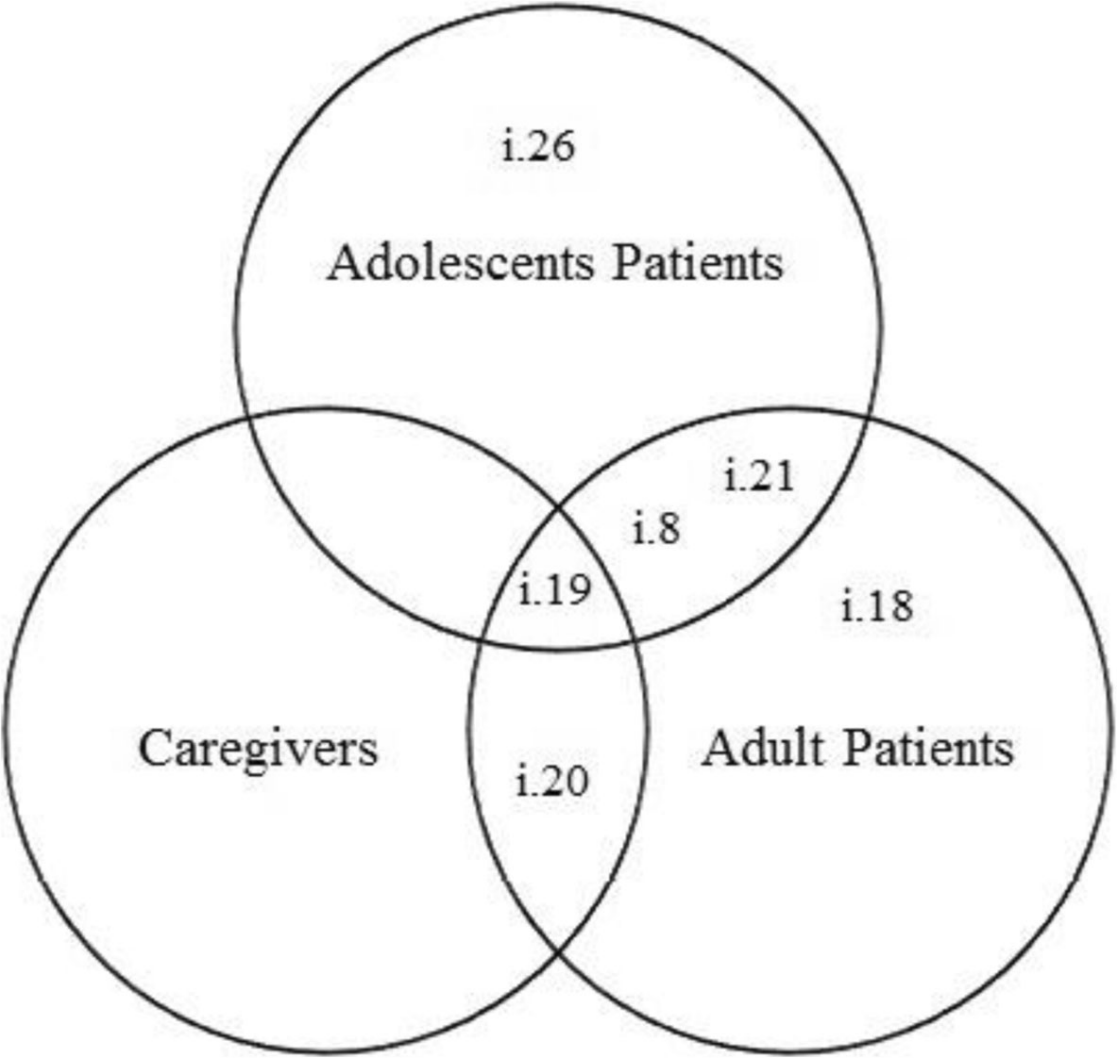
Similarities and particularities between groups according to Phenylketonuria Perceived Barrier to Treatment Inventory items mentioned by more than 50% of the participants

Despite the differences in the percentage of items endorsed in each group, more of a consensus was observed in relation to the sentences receiving the highest agreement (see Fig. 1). Thus, item 8 (i.e. “I often have no desire to follow the diet”), item 19 (i.e. “It would be easier to take the medical food if it tasted better”), item 20 (i.e. “I think the diet is very strict”), and item 21 (i.e. “I do not think I have a disease. I just need to take care of my meals”) were mentioned by 50% or more of the respondents in at least two groups. Furthermore, among adolescents item 26 (i.e. “The distance from my house to the hospital makes it more difficult to attend medical appointments”) was selected by 66.7% of this group. The same percentage of adult patients mentioned item 18 (i.e. “Sometimes I cannot resist eating foods I know are forbidden”).

### Evidence of validity: known-groups validity

To demonstrate whether a questionnaire or its items can discriminate between two groups known to differ on a variable of interest (i.e. known-groups validity), we performed the following analysis. Patients’ scores^2^ on the inventory differed across age groups (*U* = 20.000; *p* = 0.005), i.e. the adult group reported a greater number of barriers to adherence than did younger patients and their proxies. Inventory scores also differed across adherence status groups (measures of the most recent phenylalanine level) (*U* = 20.500, *p* = 0.009) (see Table 5).

**Table 5:** Differences Phenylketonuria Perceived Barrier to Treatment Inventory scores between patients’ demographic characteristics

Variable	Phenylketonuria Perceived Barrier to Treatment Inventory scores *Mdn* [*IQR*]	*Mann-Whitney test (p)*
Age group		20.500 (0.005)
Caregivers of patients 6 to 12 and patients 13 to 17 y.o. (*n* = 11)	4 [3.0–5.0]	
18 years old or older (*n* = 12)	9 [7.3–11.8]	
Current Phe Adherence^a^		20.000 (0.009)
Good (*n* = 12)	4 [3.0–8.0]	
Poor (*n* = 10)	9.5 [5.0–13.3]	
Current Median Phe Adherence^a^		33.000 (0.073)
Good (*n* = 10)	4.5 [2.8–8.5]	
Poor (*n* = 12)	7.5 [5.0–11.8]	
Sex		46.500 (0.295)
Woman (*n* = 9)	5 [3.5–8.0]	
Man (*n* = 14)	7.5 [3.8–11.3]	
Mothers’ level of education		43.000 (0.283)
4 years or less (*n* = 8)	9 [4.3–11.5]	
5 to 11 years (*n* = 15)	5 [4.0–8.0]	
Fathers’ level of education^b^		24.000 (0.185)
4 years or less (*n* = 6)	9 [4.5–11.8]	
5 to 11 years (*n* = 13)	5 [3.5–8.0]	
PKU Classification ^c^		49.500 (0.306)
Classical (*n* = 11)	8 [4.0–12]	
Mild (*n* = 12)	5 [3.3–9.5]	
Beginning of treatment		59.000 (0.948)
≤ 3 months of age (*n* = 15)	7 [4.0–10.0]	
> 3 months of age (*n* = 8)	5 [3.3–10.8]	

*Notes: PKU* Phenylketonuria. *Mdn* Median. *IQR* Interval Interquartile Range. *Phe* Phenylalanine. Current Phe level = Phe level collected prior to the study. Current Median Phe level = median of Phe level collected in the last 12 months prior to the study. Level of education = years of schooling. ^a^ One patient had no information regarding recent adherence. ^b^Four patients did not answer the question on the paternal educational achievement. ^c^ One patient with Undefined PKU type was classified as Classical PKU

When analyzing the responses of the sample divided by metabolic control, i.e. the most recent phenylalanine level collected, we found that patients considered to exhibit poor adherence agreed significantly more with items 2, 7, and 20 than did patients with good adherence. When comparing the responses of the sample divided by age group, perceptions of having questions about the disease, its causes, and its potential health impact (item 5), and the perceived high restrictiveness of the diet (item 20) were significantly more pronounced in adulthood than for adolescents and caregivers (Table 6).

**Table 6:** Frequency of agreement in each item of Phenylketonuria Perceived Barrier to Treatment Inventory according to adherence and age group

Perceived barriers (items)	Adherence status^a^			Age group		
	Good Adherence (*n* = 12) *n* (%)	Poor Adherence (*n* = 10) *n* (%)	Fisher’s Exact test *p*	Younger than 18 years old (*n* = 11) *n* (%)	18 years old or older (*n* = 12) *n* (%)	Fisher’s Exact test *p*
Patient-related factors						
1	4 (57.1)	3 (42.9)	1.000	1 (14.3)	6 (85.7)	0.069
2	1 (16.7)	5 (83.3)	**0.056**	1 (16.7)	5 (83.3)	0.155
3	1 (50.0)	1 (50.0)	1.000	0	2 (100)	0.478
4	4 (57.1)	3 (42.9)	1.000	2 (28.6)	5 (71.4)	0.371
5	2 (40.0)	3 (60.0)	0.624	0	5 (100)	**0.037**
6	1 (25.0)	3 (75.0)	0.293	1 (25.0)	3 (75.0)	0.590
7	0	4 (100)	**0.029**	1 (25.0)	3 (75.0)	0.590
8	4 (40.0)	6 (60.0)	0.391	4 (36.4)	7 (63.6)	0.414
10	1 (33.3)	2 (66.7)	0.571	1 (33.3)	2 (66.7)	1.000
Social Economic- related factors						
12	0 (0)	2 (100)	0.195	1 (33.3)	2 (66.7)	1.000
13	0 (0)	2 (100)	0.195	0 (0)	3 (100)	0.217
Treatment-related factors						
14	1 (20.0)	4 (80.0)	0.135	2 (40.0)	3 (60.0)	1.000
15	2 (40.0)	3 (60.0)	0.624	1 (16.7)	5 (83.3)	0.155
16	2 (28.6)	5 (71.4)	0.172	3 (42.9)	4 (57.1)	1.000
17	2 (40.0)	3 (60.0)	0.624	1 (20.0)	4 (80.0)	0.317
18	5 (50.0)	5 (50.0)	1.000	3 (27.3)	8 (72.7)	0.100
19	9 (52.9)	8 (47.1)	1.000	7 (38.9)	11 (61.1)	0.155
20	5 (35.7)	9 (64.3)	**0.031**	4 (26.7)	11 (73.3)	**0.009**
Condition-related factors						
21	5 (45.5)	6 (54.6)	0.670	5 (41.7)	7 (58.3)	0.684
22	3 (42.9)	4 (57.1)	0.652	2 (28.6)	5 (71.4)	0.371
Health care team and system-related factors						
23	0 (0)	2 (100)	0.195	0 (0)	2 (100)	0.478
24	1 (33.3)	2 (66.7)	0.571	1 (33.3)	2 (66.7)	1.000
25	1 (33.3)	2 (66.7)	0.571	1 (33.3)	2 (66.7)	1.000
26	3 (42.9)	4 (57.1)	0.652	5 (71.4)	2 (28.6)	0.193
27	4 (44.4)	5 (55.6)	0.666	4 (44.4)	5 (55.6)	1.000

*Notes.* Patient adherence = phenylalanine level according to the last blood exam prior to study

^a^ One patient had no information regarding recent adherence

Statistic significances are printed in bold font

### Evidence of validity: construct validity

To ascertain the construct validity of the inventory (all types of evidence support construct validity [48];), associations with criterion-related measures were tested (correlation analysis between Phenylketonuria Perceived Barriers to Treatment Inventory and disease-relevant medical markers). The frequency of perceived barriers was positively associated with recent blood Phe concentration (Kendall’s tau_b_ = 0.40; *p* = 0.011) and the median of the Phe collected 12 months prior to participating in the study (Kendall’s tau_b_ = 0.41; *p* = 0.010). It was also correlated with self-reported adherence score, as measured by the Perceived adherence - VAS (Kendall’s tau_b_ = − 0.31; *p* = 0.043): that is, an individual’s lower compliance with the behavior recommended by their health team was associated with endorsing more barriers in the inventory. In addition, correlational analysis between the Phenylketonuria Perceived Barriers to Treatment Inventory and the WASI was performed. Our hypothesis was that the instruments measure different constructs, and that deficits in intellectual and cognitive ability may lead to an increase in perceived barriers. Although, no statistically significant association was found between Phenylketonuria Perceived Barriers to Treatment Inventory score and IQ scores, the associations were in the expected direction (see Table 7).

**Table 7:** Evidences that supports construct validity

Variables	Phenylketonuria Perceived Barrier to Treatment Inventory (Score) Kendall’s tau-b test (*p*)
Phe at diagnosis (*n* = 23)	0.49 (0.749)
Recent Phe (*n* = 22)^a^	**0.40 (0.011)**
Recent Median Phe (*n* = 22)^a^	**0.41 (0.010)**
Disease knowledge -VAS (*n* = 23)	- 0.10 (0.522)
Dietary knowledge -VAS (n = 23)	0.03 (0.873)
Perceived adherence -VAS (*n* = 23)	**- 0.31 (0.043)**
Full Scale IQ (*n* = 19)^b^	- 0.23 (0.179)
Verbal IQ (*n* = 19) ^b^	- 0.13 (0.437)
Performance IQ (*n* = 19)^b^	- 0.28 (0.104)

*Notes*. *Phe* Phenylalanine. Recent Median Phe = median of the last 12 months Phe measures prior to the study

^a^ One patient had no information regarding recent adherence. ^b^Four patients did not answer WASI

Statistic significances are printed in bold font

## Discussion

The results of this study demonstrate that the Phenylketonuria Perceived Barrier to Treatment Inventory has the ability to identify perceived barriers to PKU treatment. As hypothesized, participants’ scores on the inventory were correlated with objective measures used as parameters for adherence (e.g. level of phenylalanine) and results from other applied measures that evaluated related constructs (e.g. visual analogue scales on information about the disease and its treatment, and adherence), supporting its construct validity. The higher frequency of agreement on certain inventory items among the group considered to have poor adherence provides further evidence that the instrument is capable of identifying relevant barriers to treatment. The lack of association between inventory scores and performance on cognitive tests demonstrates that answers to the inventory were not related to cognitive performance or either of the IQ test constructs.

In addition, significant differences between the group of patients under 18 years of age and those over this age show evidence of known-group validity. That is, a greater number of perceived barriers were expected to be found among adults than among younger patients, since treatment management tends to be the responsibility of individuals later in life, as other studies have observed [42].

Considering the differences between the frequencies of certain items mentioned by patients with good and poor adherence, the results point to multidimensional determinants influencing the outcomes of treatment. Thus, for some people conflicts in interpersonal relationships, as represented by the item on not talking about the diagnosis, could be associated with discomfort with disclosing their medical condition or even fear of being stigmatized—aspects of the disease mentioned by patients in previous studies [38, 41].

Another correlate of insufficient adherence was poor knowledge about what constitutes an appropriate diet, also mentioned by Bik-Multanowski et al. [34]. This can result in a menu with less food variability, thereby increasing the chances of the patient ending up consuming food that is not allowed. A perception that their food options are greatly limited can also lead to patients over-consuming foods that require controlled intake, resulting in non-voluntary non-adherence. Such lack of information can contribute to a low variety of dietary foods being used, which in turn may give rise to the perception that the diet is much more restrictive than it actually is. On the other hand, that food options are limited is also a reality. Whether it is food prepared at home, ready meals, or menus offered in restaurants, as other studies have shown patients perceive there to be a scarcity of options [49], and with individuals presenting other forms of food restriction [50, 51], this has an impact on their treatment.

Regarding the differences between the age groups on agreement with the inventory items, one possible explanation is the role of the main person responsible for managing the patient’s treatment. For example, barriers such as having questions about their disease and the health consequences of non-adherence and perceiving there to be few available food options were not mentioned any of the adolescent participants. Thus, it is possible that the absence of such issues among younger people is associated with the fact that decisions about their treatment are still the responsibility of their caregivers.

Another result that may demonstrate the lower involvement of adolescents in decisions about their treatment is the high incidence of agreement in this group to the item concerning distance from the treatment center as a barrier. Di Ciommo et al. [37] mentioned that adherence behavior tends to be linked to strength of habit rather than a learned association between wrong choices and harmful consequences, and thus may result in a difficulty in understanding the requirements of the treatment.

However, in addition to obtaining the information necessary to understand their treatment requirements, patients’ psychosocial and emotional factors may have a role in the process of turning knowledge into healthy attitudes [52, 53]. According to our study there was no association between perceived barriers to treatment and self-rated levels of knowledge about the disease and dietary treatment, suggesting that knowledge alone is not sufficient to improve adherence to phenylketonuria treatment.

Psychosocial factors may also have influenced the lack of relationship between levels of phenylalanine found in this study and dissatisfaction with the taste of medical food. This complaint is a well-known challenge in the literature [16, 36, 39, 40, 43], and although this item was mentioned often, agreeing with it was not associated with poor adherence to treatment.

The results presented here must be interpreted in light of some limitations. The most obvious is the restricted generalizability of the findings because of the small sample size and the fact that participants volunteered to take part in the study. Although, the final sample size in the study can not assure representativeness, we believe the characteristics of the sample included are heterogeneous enough to produce relevant information about perceived barriers. In addition, the lack of direct patient input in the early stages of the creation of the instrument, and combining responses from self-report (adolescents 13–17) and proxy respondents (caregivers of children 6–17) in a single group in comparisons shown in Tables 5 and 6, may be seen as limitations. Further, the study do not include proxy-report regarding children below 6 years old, or adults above 35, future studies may try to address this population. Lastly, Phe levels in blood—our measure of adherence to treatment—is not free from bias. Even when using values considered to be more objective, such as biomarkers, previous studies have shown that it is possible for the phenylketonuria patient to make changes to his or her diet before blood collection [35, 54].

All things considered, the encouraging data presented here are original and innovative. We expect Phenylketonuria Perceived Barrier to Treatment Inventory to be useful in future research addressing barriers related to phenylketonuria treatment; as an outcome assessment for evaluating interventions aimed at reducing perceived barriers; and/or for training to involve patients or caregivers more actively in meal preparation or self care to achieve better adherence to treatment.

## Conclusions

The results of this study suggest that the Phenylketonuria Perceived Barrier to Treatment Inventory has the ability to identify perceived barriers to PKU treatment. Preliminary evidence of validity suggest that the inventory may be useful in future research addressing barriers related to phenylketonuria treatment; as an outcome assessment for evaluating interventions aimed at reducing perceived barriers; and/or for training to involve patients or caregivers more actively in meal preparation or self care to achieve better adherence to treatment. These results support the need for further research on barriers perceived by PKU patients.

## Supplementary information

**Additional file 1.**

## Data Availability

The data that support the findings of this study are available from the corresponding author upon reasonable request.

## Abbreviations

PKU: Phenylketonuria
PAH: Phenylalanine hydroxylase
Phe: Phenylalanine
VAS: Visual analogue scales
WASI: Wechsler Abbreviated Scale of Intelligence
IQ: Intellectual and cognitive ability

## Footnotes

1 The visual analogue scales items were translated from the Brazilian Portuguese to English to the present paper.

2 Where patients are younger than 13 years old, scores correspond to the caregiver version of the inventory (proxy report).
