# Supplementary material for: Development of an inventory to assess perceived barriers related to PKU treatment
Source: J Patient Rep Outcomes. 2020 May 1;4:29. doi: 10.1186/s41687-020-00194-w (PMC7195505; doi:10.1186/s41687-020-00194-w)
Supplement: Supplementary file 1 — Additional file 1. [file 41687_2020_194_MOESM1_ESM.docx]

SUPPLEMENTARY MATERIAL

During data collection phase, patients were allowed to add a particular perceived barrier to treatment that was not listed in the present final version of the inventory. The inventory included, at end of the instrument, an open-ended question allowing the participant to describe a particular perceived barrier to treatment that was not listed in the inventory.

Bellow, find the information collected. Some of the information collected (26,3%) were already covered by the inventory.

| Translation in English  (The information was translated from the Brazilian Portuguese to English to the present paper) | Original in Portuguese | (Number) Inventory item that addresses the topic. |
| --- | --- | --- |
| Open question at the end of inventory# | Pergunta aberta ao final do inventário |  |
| Now make a list of up to five barriers (starting with the most important) that most hinder you from adhering to treatment, which has not been mentioned before. | Agora, faça uma lista com até cinco barreiras (começando pela mais importante) que mais atrapalham para a adesão ao tratamento, que não tenham sido mencionadas anteriormente. |  |
| **Caregiver Group’s answers** | | |
| *Failure in monthly public delivery of Metabolic Formula; Lack of Metabolic Formula.* | *Falta da entrega da Fórmula Metabólica em alguns meses; Falta da Fórmula Metabólica.* | (27) There are months when the formula is not delivered. |
| *Limited option of food; Lack of free supply of special foods (low in phenylalanine)*; *Food in the supermarket. We have no easy access to special foods; High cost of these foods; Buy special foods for the diet; Difficulty access to diet products; Lack of available hypoproteics foods.* | *Pouca opção de comidas; Não fornecer os alimentos (tipo farinha e outros derivados); Alimentos em supermercado. Não tem facilidade no acesso; Alto custo dos alimentos [especiais]; Comprar alimentos especiais para a dieta; Dificuldade de acesso aos produtos para a dieta; Falta de alimentos hipoproteicos.* | (20) I think the diet is very strict. If he/she could eat a broader range of food, it would be easier. |
| *The location of the town where we live.* | *Localização da cidade onde residimos.* | (26) The distance from our house to the hospital makes it more difficult to attend the medical appointments. |
| *The lack of knowledge about the disease [of the health professional who provided the diagnosis].* | *A falta de informação inicial [do profissional que forneceu o diagnóstico].* |  |
| *Dissemination of PKU treatment.* | *Divulgação ao tratamento.* |  |
| *The absence of genetics specialists available in our city.* | *Não ter especialistas de genética na minha cidade.* |  |
| *Lack of pedagogy in schools.* | *Falta de pedagogia nas escolas.* |  |
| *The first impact when I was disclosed about the disease in my child.* | *O choque inicial ao saber da doença.* |  |
| *Concern about the child's awareness of treatment when he becomes main responsible for the diet.* | *Preocupação sobre a conscientização do filho em relação ao tratamento.* |  |
| *Process of feedback to participants on treatment-related research.* | *Retorno ao paciente sobre pesquisas relacionadas ao tratamento.* |  |
| *The constant exchange of Formula brand which is provided to us in a short time.* | *Troca constante de Fórmula em curto períodos.* |  |
| *We have not yet obtained free sapropterin treatment from government.* | *Não ter conseguido ainda o medicamento sapropterina via judicial.* |  |
| **Adult Patient Group’s answers** | | |
| *Not being able to go to parties.* | *Não poder ir às festas.* | (4) Although I feel like going, I don’t go to some parties or family events because I know that there will be food that I cannot eat. |
| *Consumption of foods that are controlled or not allowed; Consumption of alcohol; Frequent consumption of soda.* | *Consumo de alimentos controlados [em excesso] ou inadequados; Consumo de bebida alcoólica; Uso frequente de refrigerante.* | (18) Sometimes I cannot resist and I eat foods I know are forbidden. |
| *Sometimes it bothers me that people ask me what I'm eating.* | *Às vezes me incomoda que as pessoas me perguntem sobre o que eu estou comendo.* |  |
| *Others have difficulty in understanding the disease.* | *Dificuldade dos outros em entenderem [a doença].* |  |
| *Friends influence me to get off the diet in some situations.* | *Influência de amigos a sair fora do tratamento.* |  |
| *When I go to a restaurant, for example, and there are not many [low phenylalanine] meal options.* | *Quando, no local, por exemplo, restaurante, lanchonete, não tem muitas alternativas de alimentos.* |  |
| *Sometimes taking care of my brother's meals (who also has PKU) ends up disrupting my own meals.* | *Às vezes cuidar das refeições do meu irmão (que também tem PKU) acaba atrapalhando eu fazer as minhas próprias refeições adequadamente.* |  |

# Note: there were no answers in the Adolescents’ Group
